# Disulfidptosis decoded: a journey through cell death mysteries, regulatory networks, disease paradigms and future directions

**DOI:** 10.1186/s40364-024-00593-x

**Published:** 2024-04-29

**Authors:** Jinyu Chen, Boyuan Ma, Yubiao Yang, Bitao Wang, Jian Hao, Xianhu Zhou

**Affiliations:** The Second Affiliated Hospital, Guangzhou Medical University, Guangzhou, 510260 China

**Keywords:** Disulfidptosis, Ferroptosis, SLC7A11, Pentose phosphate pathway(PPP), Nicotinamide Adenine Dinucleotide Phosphate (NADPH), Cystine Cysteine RAC1-WRC pathway, Glucose starvation

## Abstract

Cell death is an important part of the life cycle, serving as a foundation for both the orderly development and the maintenance of physiological equilibrium within organisms. This process is fundamental, as it eliminates senescent, impaired, or aberrant cells while also promoting tissue regeneration and immunological responses. A novel paradigm of programmed cell death, known as disulfidptosis, has recently emerged in the scientific circle. Disulfidptosis is defined as the accumulation of cystine by cancer cells with high expression of the solute carrier family 7 member 11 (SLC7A11) during glucose starvation. This accumulation causes extensive disulfide linkages between F-actins, resulting in their contraction and subsequent detachment from the cellular membrane, triggering cellular death. The RAC1-WRC axis is involved in this phenomenon. Disulfidptosis sparked growing interest due to its potential applications in a variety of pathologies, particularly oncology, neurodegenerative disorders, and metabolic anomalies. Nonetheless, the complexities of its regulatory pathways remain elusive, and its precise molecular targets have yet to be definitively identified. This manuscript aims to meticulously dissect the historical evolution, molecular underpinnings, regulatory frameworks, and potential implications of disulfidptosis in various disease contexts, illuminating its promise as a groundbreaking therapeutic pathway and target.

## Introduction

Cell death plays an important role in many life processes, including embryonic development and the expulsion of damaged, infected, or superfluous cells [1–3]. Furthermore, it serves an important role in coordinating immune responses and regulating autoimmunity. Cell death is broadly classified into two types: Programmed Cell Death (PCD) and Accidental Cell Death (ACD) [4–7]. ACD is an unregulated cell death that is typically triggered by harmful stimuli [5]. In contrast, PCD refers to a variety of genetically regulated modes of cell death that are essential for maintaining internal homeostasis [5]. PCD is essential for embryonic development and has a significant impact on the onset and progression of many diseases [6, 7].

To date, the scientific community has identified several forms of PCD, including apoptosis, ferroptosis, necroptosis, and pyroptosis, among others [2–7]. Liu et al. have discovered a new type of Programmed Cell Death, termed disulfidptosis [8]. This process is especially noticeable during glucose deficiency when inhibition of the Pentose Phosphate Pathway (PPP) causes a deficit in Nicotinamide Adenine Dinucleotide Phosphate (NADPH). This causes cystine to accumulate in cells that express high levels of SLC7A11. The confluence of cystine accumulation and NADPH scarcity causes increased disulfide stress within cells. This stress causes the formation of numerous disulfide bonds within F-actin, which causes it to contract and detach from the cell membrane, eventually leading to cell death [8, 9]. In this complex process, the RAC1-WRC pathway plays an important role. Overexpression of RAC1 activates NCKAP1, resulting in the assembly of the WAVE Regulatory Complex (WRC), which includes subunits such as WAVE-2, CYFIP1, Abi2, and HSPC300. This assembly may exacerbate the formation of disulfide bonds within F-actin. It accomplishes this by promoting Arp2/3-mediated lamellipodia formation, which is a critical step in disulfidptosis [8, 10–13].

Disulfide stress is a form of oxidative stress that can cause abnormal disulfide bonds between the thiol groups of active cysteine residues in redox-sensitive proteins [14]. The relationship between disulfide stress and disulfidptosis lies in the fact that disulfide stress is one of the triggering factors leading to disulfidptosis. When cells are exposed to conditions that disrupt the balance of disulfide bonds, if the imbalance cannot be effectively restored through the cell’s internal stress response mechanisms, prolonged or extreme disulfide stress may lead to cell death through the pathway of disulfidptosis [8, 9]. This process may involve specific molecular pathways and signal transduction mechanisms, such as the activation of the protein folding surveillance system, the depletion of antioxidant defense mechanisms, and the dysfunction of intracellular disulfide bond-related enzymes (such as protein disulfide isomerase) [15–17].

As research into the mechanisms of disulfidptosis continues, a growing body of evidence suggests a link to oncological conditions, neurological disorders, and bone metabolism abnormalities [18–46]. As a result, a thorough investigation of disulfidptosis mechanisms has significant clinical implications for the understanding and treatment of these conditions. This article attempts to provide an in-depth review of research advances in disulfidptosis. It provides an overview of its discovery, the complex regulatory networks that influence it, such as amino acid metabolism, NADPH metabolism, and the RAC1-WRC pathway as well as its potential roles in various diseases. It also highlights the unanswered questions and challenges in this domain.

## Historical overview of disulfidptosis discovery

The transport of intracellular materials is critical for maintaining physiological functions and controlling biological processes. In this regard, the discovery of the Solute Carrier (SLC) family of transport proteins has been critical. Their critical roles in facilitating the transport of glucose, amino acids, and metal ions provide essential insights into complex intracellular dynamics [10].

**Fig. 1:**
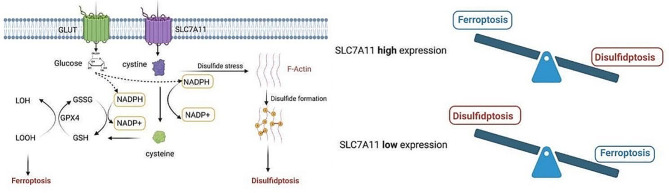
**a** Interaction network diagram of ferroptosis and disulfidptosis-induced cell death **b** The model of SLC7A11-mediated cell death: interplay between disulfidptosis and ferroptosis

Initial research highlighted the importance of the Solute Carrier family member SLC7A11, particularly in maintaining intracellular glutathione (GSH) levels and preventing cell death caused by oxidative stress [47]. Furthermore, studies have shown that cystine deprivation or the impediment of cystine uptake, as mediated by SLC7A11, can initiate the ferroptosis process [48, 49] (Fig. 1a). This phenomenon involves glutathione (GSH) depletion caused by cystine scarcity or SLC7A11 inactivation, which results in reduced functionality of GSH-dependent glutathione peroxidase 4 (GPX4). This reduction, in turn, causes an excess of reactive oxygen species (ROS) to accumulate, resulting in iron-induced cell death [49](Figure 1a).

However, recent research has provided a different perspective. It suggests that when glucose levels are low, increased expression of SLC7A11 paradoxically accelerates cell death [8–13, 50–52]. For example, in glioblastoma cells starved for glucose, cystine uptake via xCT (with SLC7A11 as the catalytic subunit) leads to NADPH depletion, ROS accumulation, and cell death [50]. Moreover, a pivotal 2020 study by Liu Xiaoguang et al., published in ‘Nature Cell Biology,’ revealed that under glucose starvation, cells with elevated SLC7A11 expression and cystine accumulation are prone to cell death. This process, however, can be slowed by inhibiting the formation of disulfides [9]. Their research identified a new type of cell death called ‘Disulfidptosis’. The actin cytoskeleton’s sensitivity to disulfide stress is critical in this process. Disulfidptosis is distinguished by atypical disulfide bond formation within the actin cytoskeleton. This causes F-actin to contract and eventually detach from the cell membrane. Such changes result in morphological alterations and cell death in glucose-deprived cells with elevated SLC7A11 expression [8] (Fig. 1a).

SLC7A11 has two distinct functions in disulfidptosis and ferroptosis, highlighting its complex role in modulating cellular redox equilibrium and determining the balance of cell survival and death [52]. Ferroptosis and disulfidptosis [11, 52] may interact, with SLC7A11 potentially playing a critical role (Fig. 1a and b). SLC7A11 is a cystine transporter responsible for the uptake of intracellular cystine, a sulfur-containing amino acid [53, 54]. High expression of SLC7A11 leads to excessive cystine uptake, which, following glucose starvation, results in NADPH depletion and the loss of the ability to reduce intracellular disulfide bonds. This triggers the formation of abnormal disulfide bonds in specific proteins, especially those related to the cytoskeleton like F-actin. These abnormal disulfide bonds cause the collapse and dysfunction of the F-actin cytoskeleton, ultimately leading to disulfidptosis [8–13]. High expression of SLC7A11, by promoting the import of cystine and increasing the synthesis of glutathione (GSH), maintains intracellular antioxidant defense. GSH, as a cofactor for glutathione peroxidase 4 (GPX4), aids in reducing lipid peroxides, preventing the accumulation of lipid peroxidation, and thus inhibiting ferroptosis [52].(Fig. 1a) In essence, under conditions of glucose starvation, cells with high expression of SLC7A11 undergo disulfidptosis; whereas, under the same conditions, cells with low expression of SLC7A11 are less prone to disulfidptosis. High expression of SLC7A11 inhibits ferroptosis, while low expression of SLC7A11 promotes ferroptosis.(Fig. 1b) These findings not only improve our understanding of disulfidptosis and its implications in a variety of diseases, but also pave the way for new research directions and therapeutic possibilities.

## The regulatory network of disulfidptosis

The onset of disulfidptosis is attributed to a combination of factors. Key among these are cystine accumulation, NADPH depletion, and WRC assembly, all of which collectively contribute to the formation of disulfide bonds within F-actin [8, 10–13]. To better understand the complexities of disulfidptosis, this paper examines the regulatory influences exerted by these three important aspects of the disulfidptosis process.

### Cystine metabolism

Cystine metabolism plays an important role in a variety of biological processes, notably including protein synthesis, glutathione production, and the maintenance of intracellular redox equilibrium [55–60]. The accumulation of cystine causes disulfide stress, which is a critical catalyst in the initiation of disulfidptosis [9]. As a result, a thorough understanding of cystine metabolism’s regulatory function in disulfidptosis is imperative. This section will look at the transporters involved in cystine metabolism, such as xCT, cystine transporter, cysteine transporter, and glutamate transporter. Furthermore, it will investigate the role of the cystine-cysteine cycle and glutathione metabolism in the context of disulfidptosis.

#### Transporters related to cystine metabolism

##### Cystine/glutamate antiporter (xCT)

**Fig. 2:**
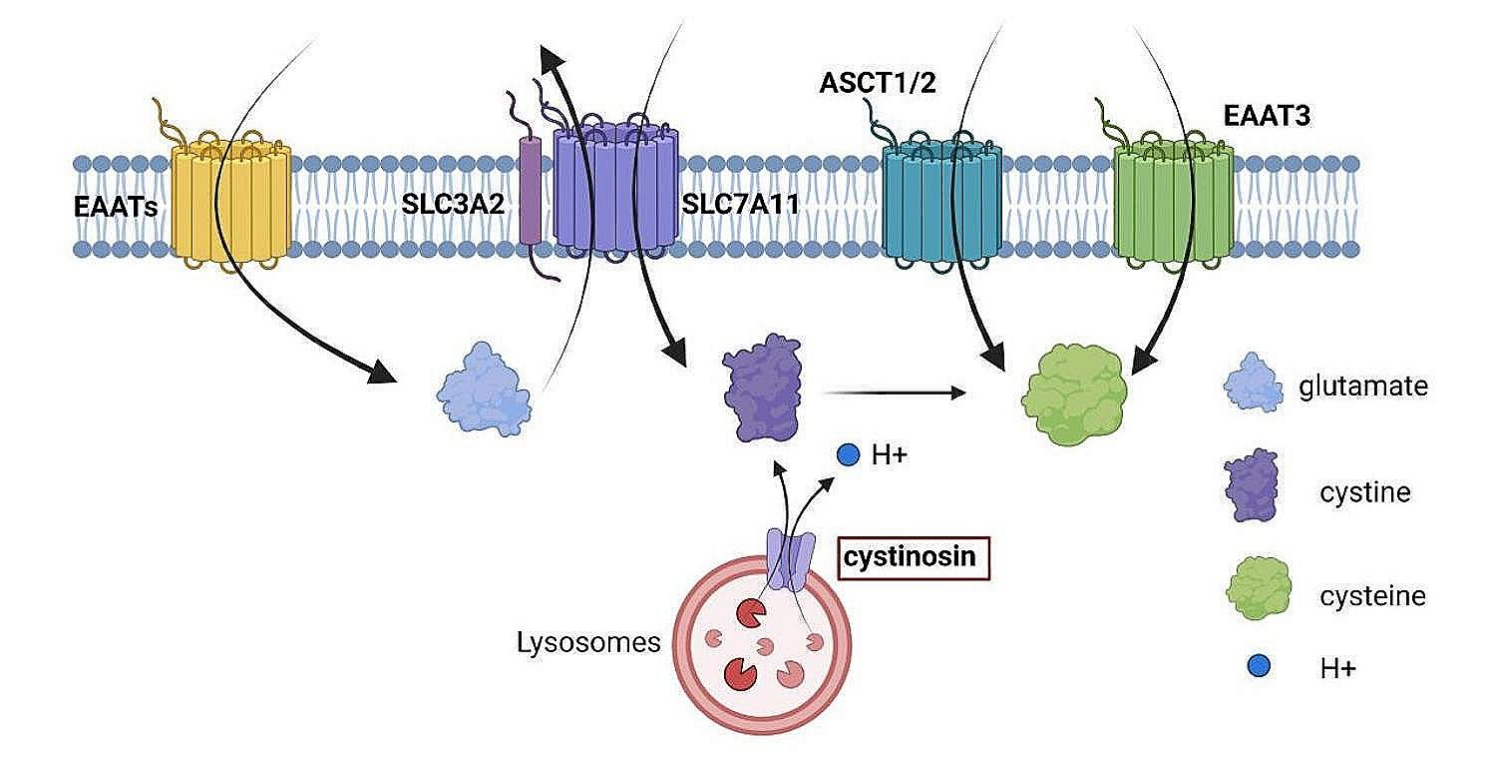
Transporters associated with cystine metabolism: cystine transporter, cysteine transporter, and glutamate transporter

The process of disulfidptosis relies heavily on xCT [8, 9]. As a Na-independent anionic amino acid transport system, xCT distinctively facilitates the transport of cystine into the cell and glutamate out of it, a crucial exchange between cystine and glutamate [53, 54] (Fig. 2). The xCT transporter is made up of two subunits: SLC7A11, which controls antiporter activity, and SLC3A2, which is important for protein stability [61–65]. In cells with elevated SLC7A11 expression, NADPH depletion causes cystine accumulation, which initiates disulfidptosis [8]. Inhibitors of xCT, such as glutamate, erastin, sulfasalazine, and sorafenib, can block cystine uptake, thereby disrupting GSH synthesis and causing iron-dependent oxidative cell death [49, 56], but the precise effects of these inhibitors on disulfidptosis require further investigation. The expression level of SLC7A11 influences not only disulfidptosis but also ferroptosis, implying a possible link between these two types of cell death [8, 10–13] (Fig. 1b).

##### Cystinosin

Cystinosin, a lysosomal transmembrane protein encoded by the CTNS gene, facilitates the co-transport of cystine and H + in a 1:1 ratio, enabling cystine transfer from the lysosome to the cytoplasm [66] (Fig. 2). Most amino acids are typically found in lower concentrations in lysosomes than in the cytoplasm in proliferating cells. Cystine, on the other hand, is a notable exception, with concentrations in the lysosomes significantly exceeding those in the cytoplasm [67]. Cystine, once transported to the cytoplasm by cystinosin, can be reduced to cysteine. This cysteine then contributes to the synthesis of the antioxidant, glutathione [68]. This mechanism has the potential to influence the cystine-cysteine cycle and thus the disulfidptosis process.

Cystinosin dysfunction causes abnormal cystine accumulation within lysosomes, which leads to cystinosis [69–71]. In this pathological state, the abnormal accumulation of cystine causes the formation of intracellular crystals, gradually inflicting damage upon cells and organs [72]. Cysteamine, a therapeutic agent used to treat cystinosis, aids in the removal of cystine from lysosomes [73]. Cysteamine, which enters lysosomes through an unidentified transporter, facilitates the breakdown of cystine into cysteine and cysteine–cysteamine disulfide. These breakdown products are then exported from the lysosome via the cysteine and cationic amino-acid transporters [74, 75]. Cysteamine has been shown to reduce and postpone the complications associated with nephropathic cystinosis, thereby increasing the life expectancy of affected patients [76]. However, it is unclear abnormal cystinosin activation or cysteamine application influences the abnormal cytoplasmic accumulation of cystine, potentially triggering disulfidptosis, and this hypothesis remains to be rigorously tested. Therefore, future research is essential to empirically investigate these potential mechanisms and their implications.

##### Cysteine transporter

Cysteine is transported intracellularly by specialized transporters, most notably the Alanine-Serine-Cysteine Transporters 1 and 2 (ASCT1/2) and the Excitatory Amino Acid Transporter 3 (EAAT3) [60] (Fig. 2). These transporters directly facilitate tcysteine transport from the extracellular milieu into the cell, playing an essential role in sustaining the intracellular amino acid equilibrium.

###### ASCT1

ASCT1, a member of the SLC family encoded by the SLC1A4 gene, is widely distributed throughout the human body. This includes the skeletal muscles, lungs, kidneys, ovaries, heart, and the gastrointestinal tract [77]. According to research, ASCT1 plays an important role in cysteine reuptake within the brain [78].

###### ASCT2

The SLC1A5 gene encodes ASCT2, which belongs to the SLC family [79]. ASCT2 has a high affinity for a variety of amino acids, including L-Alanine, L-Serine, L-Cysteine, L-Threonine, and L-Glutamine. Its transport activity is determined by the intracellular concentration gradients of these specific amino acids [80]. ASCT2 is commonly overexpressed in a variety of proliferating cancer cells, including hepatocellular carcinoma, non-small cell lung cancer, colorectal cancer, gastric cancer, and clear cell renal cell carcinoma [81–86]. This overexpression is thought to accommodate the cancer cells’ increased glutamine requirements. While ASCT2’s cysteine transport is not highly specific, its potential role in cysteine accumulation and the subsequent induction of disulfidptosis requires further investigation.

###### EAATs

The Excitatory Amino Acid Transporter (EAAT) family consists of five distinct transport proteins, each with unique expression patterns in neurons and glial cells throughout the Central Nervous System [87]. EAAT1 and EAAT2 are predominantly expressed in astrocytes, whereas EAAT3, EAAT4, and EAAT5 are primarily found in neurons. Notably, EAAT3 has the unique ability to transport extracellular cysteine into neurons, which is a crucial rate-limiting step in the synthesis of neuronal glutathione [88, 89]. Each member of the EAAT family has glutamate transporter activity, which is essential for sustaining proper glutamatergic signaling and avoid the potentially toxic accumulation of this amino acid in the extracellular space [87] (Fig. 2). EAAT4 and EAAT5 also act as glutamate-gated chloride ion channels [90].

ASCT1/2 and EAAT3 have been identified as transporters capable of translocating extracellular cysteine into the cell interior [60]. Existing research has shown that thiol oxidants, such as diamide and diethyl-maleate, can facilitate the conversion of intracellular cysteine to cystine, exacerbating intracellular disulfide stress [8, 9]. However, it remains unclear whether the overactivation of ASCT1/2 and EAAT3 under the influence of thiol oxidants can mimic the accumulation of cystine seen with high expression of SLC7A11, thereby promoting the occurrence of disulfidptosis.Definitive evidence for this association is still lacking, highlighting the importance of investigating this mechanism and providing clear guidance for future research directions.

#### The cystine-cysteine cycle

The dynamic cycling mechanism between cystine and cysteine is critical in the regulation of disulfidptosis, particularly in the presence of the thioredoxin system [46]. Cystine is predominantly transported into the cell by SLC7A11 and then reduced to cysteine, a process that requires the presence of NADPH [8, 9]. During this process, the thioredoxin system modulates the protein disulfide/dithiol balance through disulfide reductase activity, which is critical for oxidative stress defense [91, 92]. Because of its reduced thiol group, cysteine quickly oxidizes to cystine within the oxidatively charged extracellular milieu [9]. Furthermore, activators such as disulfide-reducing agents, thiol compounds, and thiol oxidants, influence disulfidptosis by modulating the cystine-cysteine cycle [8, 9].

##### Thioredoxin system

**Fig. 3:**
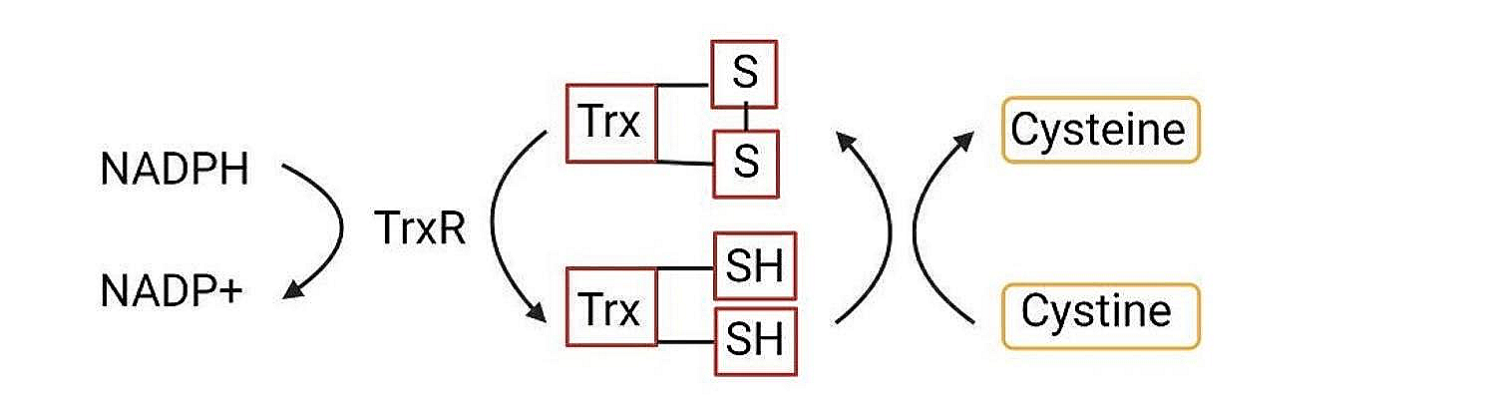
Thioredoxin system regulation of cysteine metabolism

The thioredoxin (Trx) system, which includes NADPH, thioredoxin reductase (TrxR), and thioredoxin, is an important antioxidant mechanism that counteracts oxidative stress via disulfide reductase activity [93]. Importantly, the Trx system regulates the cystine/cysteine balance, which helps to prevent disulfidptosis caused by cystine accumulation [46]. TrxR uses NADPH, which is produced via the PPP, to sustain Trx in its reduced form, facilitating the continuous reduction of cystine to cysteine [94, 95] (Fig. 3). In mammals, three distinct TrxR isoforms have been identified: TRXR1 in the cytoplasm, TRXR2 in the mitochondria, and TRXR3, which is only expressed in testes. Similarly, mammalian cells contain two types of Trx: Trx1 in the cytoplasm and Trx2 in mitochondria.

**Fig. 4:**
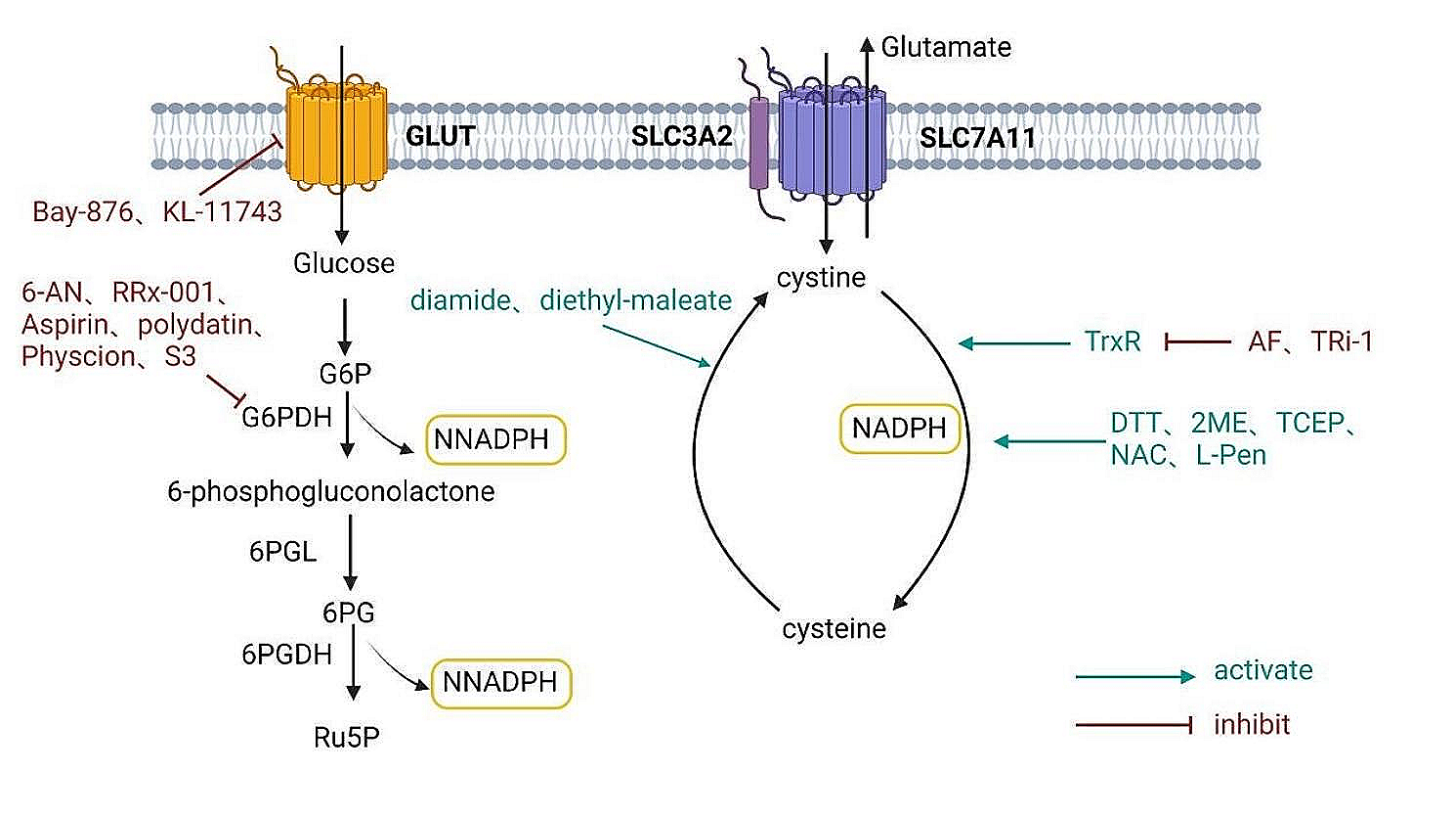
Regulatory role of inhibitors and activators in disulfidptosis

The thioredoxin system is essential for a variety of cellular functions, including antioxidant defense, cell cycle regulation, and gene transcription control, which all influence cellular growth and survival pathways [96]. Recent years have seen significant advances in the study of TrxR inhibitors, which include a wide range of compounds such as metal complexes, Michael acceptors, arsenic and selenium derivatives, and nitro(hetero)aromatic compounds [97]. However, the role of these inhibitors in regulating disulfidptosisis unknown, suggesting a promising avenue for future research. For example, Auranofin (AF), a TRXR1 inhibitor, restricts not only TRXR1 activity but also TRXR2, glutathione S-transferases, and various phosphatases. In contrast, TRXR1-specific inhibitor (TRi-1) directly targets TRXR1 [98–101]. Studies have demonstrated that osteoclast precursors are more sensitive to Auranofin (AF) and TRi-1 than bone marrow-derived monocytes. These inhibitors significantly reduce the disulfide reduction rate in osteoclast precursors, which improves cystine transport and leads to cystine accumulation. This accumulation causes increased cellular disulfide stress, which culminates in disulfidptosis [46] (Fig. 4).

Although current literature is limited to osteoclast precursors, this mechanism highlights the crucial role of the thioredoxin system in regulating the balance of intracellular disulfide bonds. In pathological states such as cancer, cardiovascular diseases, and neurodegenerative diseases, abnormal regulation of the thioredoxin system is closely associated with the onset and progression of diseases [102–104]. In cancer cells, the activation of the thioredoxin system can promote tumor growth and survival, inhibiting apoptosis, thereby facilitating the development of cancer [105, 106]. In cardiovascular diseases, alterations in the thioredoxin system may affect the oxidative stress response, inflammatory response, and endothelial function of cardiomyocytes, thereby participating in the formation of diseases such as atherosclerosis and myocardial infarction [107, 108]. In the case of neurodegenerative diseases, the thioredoxin system plays a key role in protecting neuronal cells from oxidative damage and maintaining neural system functions [109, 110]. Given the critical role of the thioredoxin system in these diseases, we speculate that TRXR1-specific inhibitors, by affecting the thioredoxin system and thus disulfide bonds, may also be applicable to cancer, cardiovascular diseases and CNS diseases. Future research is expected to provide more evidences to further confirm this point, offering a theoretical basis for the application of the thioredoxin system in disease treatment.

##### Activator

In addition to the thioredoxin system, the cystine-cysteine cycle is regulated by a number of other activators. Several compounds, including 2-deoxyglucose (2DG), disulfide reductants like dithiothreitol (DTT), β-mercaptoethanol (2ME), or tris(2-carboxyethyl)phosphine (TCEP), and thiol compounds like N-acetylcysteine (NAC) and L-penicillamine (L-Pen), can prevent cancer cells with high SLC7A11 expression from dying glucose starvation [8, 9] (Fig. 4). Specifically, 2DG supplies NADPH to disulfide reductases. Compounds like DTT, 2ME, and TCEP directly convert cystine to cysteine. Meanwhile, NAC and L-Pen promote the regeneration of free thiols via disulfide exchange mechanisms [8, 47]. These pharmacological agents effectively inhibit disulfidptosis in cancer cells by preventing cystine accumulation [8–13]. Recent research indicates that NAC, L-Pen, and 2ME can effectively reduce intracellular cystine levels and prevent disulfidptosis in osteoclast precursors, especially after TRXR1 inhibition [46]. Thiol oxidants, such as diamide and diethyl-maleate, on the other hand, exacerbate disulfide stress, hastening cell death in those with elevated SLC7A11 expression during glucose deprivation [8–13] (Fig. 4).

In conclusion, cysteine and its metabolic processes play a critical role in both physiological and pathological states of cells. In the study of disulfidptosis, the cystine uptake mediated by SLC7A11, the NADPH-dependent reduction process, and the regulatory role of the thioredoxin system are critical for understanding this complex phenomenon. These mechanisms shed light on cellular responses to oxidative stress and offer potential targets for novel therapeutic interventions against cancer and other oxidative stress-related diseases.

#### Glutathione metabolism

Glutathione metabolism is crucial for maintaining redox balance, especially, in regulating ferroptosis and disulfidptosis. As the most abundant low-molecular-weight thiol in cells, glutathione also serves as the most important low-molecular-weight antioxidant [111]. GSH is essential for many cellular processes, primarily by effectively scavenging free radicals and other reactive oxygen species such as hydroxyl radicals, lipid peroxyl radicals, peroxynitrite, and H_2_O_2_. This detoxification occurs through both direct chemical reactions and enzyme-catalyzed pathways [112].

**Fig. 5:**
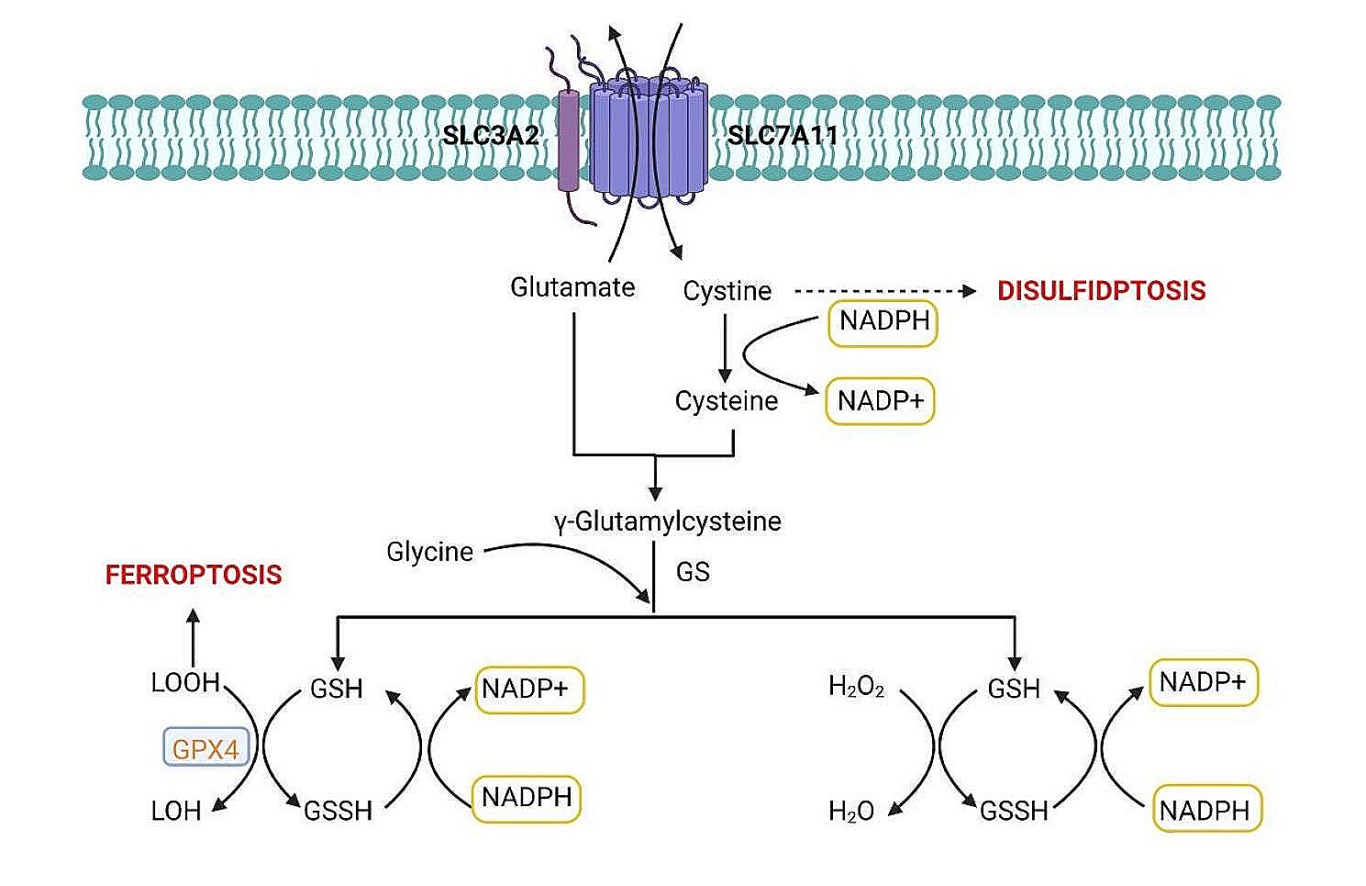
Glutathione Metabolism: SLC7A11, a catalytic subunit of system XcT, absorbs cystine from the extracellular environment and converts it to cysteine, which is then transformed into γ-glutamylcysteine in the presence of γ-GCS by binding with glutamate. This process consumes NADPH and converts it into NADP+. GS facilitates the addition of glycine to γ-glutamylcysteine, resulting in GSH synthesis. In the context of ferroptosis, which is caused by high levels of LOOH in cell membranes, reduced GSH plays an important antioxidant role. GPX4 uses GSH to reduce LOOH to LOH, preventing lipid hydroperoxides and inhibiting ferroptosis. Concurrently, GSH is oxidized to GSSG. The GSSG is then converted back to GSH via a reduction reaction mediated by GR, consuming NADPH in the process. Continuous detoxification of H_2_O_2_ by GSH also requires the consumption of NADPH.γ-GCS: γ-glutamylcysteine synthetase; GS: glutathione synthetase; GSH: glutathione; GPX4: Glutathione peroxidase 4; LOOH: lipid hydroperoxides; LOH: lipid alcohols; GSSG: oxidized glutathione; GR: glutathione reductase

High SLC7A11 expression promotes GSH synthesis and activates GPX4, which converts lipid hydroperoxides (LOOH) into lipid alcohols (LOH), preventing excessive lipid peroxidation and inhibiting ferroptosis [113] (Fig. 5). In cells with high SLC7A11 expression and cystine uptake, H_2_O_2_ treatment causes excessive intracellular accumulation of cystine and other disulfide molecules, as well as NADPH depletion. This overloads GSH’s beneficial role, resulting in a rapid onset of disulfidptosis [114] (Fig. 5).

Furthermore, NADPH is important in glutathione metabolism, as it is required for GSH synthesis. However, NADPH depletion may also contribute to disulfidptosis, a hypothesis that requires further investigation. Thus, under glucose starvation, the occurrence of disulfidptosis in SLC7A11-high cells may be due to more than one factor. H_2_O_2_-induced cell death in these cells could also be classified as disulfidptosis, which is primarily caused by NADPH depletion [114]. Although SLC7A11-positive cells may resist ferroptosis, they may become susceptible to disulfidptosis once NADPH is depleted.

Overall, glutathione metabolism regulates both ferroptosis and disulfidptosis. These processes highlight the importance of cystine accumulation and NADPH depletion in cell death mechanisms. Such discoveries not only enhance our understanding of cellular metabolic processes and cell death but also provide new opportunities for therapeutic interventions, particularly in cancer treatment.

### NADPH metabolism

Disulfidptosis is a unique type of cell death that is intricately linked to glucose deprivation and NADPH depletion. Notably, metabolic dysregulation of NADPH emerges as a hallmark of disulfidptosis, emphasizing the importance of maintaining NADPH homeostasis as a strategy for combating this cell death mode. Several metabolic pathways and enzymes regulate NADPH homeostasis, with the pentose phosphate pathway (PPP) playing a criticalrole in disulfidptosis [8].

#### Sources of NADPH

NADPH is an essential component of intracellular energy metabolism, playing an important role in maintaining the cellular redox balance [115]. Glycolysis and oxidative phosphorylation are the primary processes responsible for converting nutrients into energy that is required for various cellular functions [116]. NADPH depletion caused by glucose starvation is a critical step in disulfidptosis [8, 10–13]. Therefore, understanding the NADPH production and consumption pathways is critical for gaining a thorough understanding of disulfidptosis. The metabolic pathways and enzymes listed below are primarily responsible for NADPH homeostasis [117, 118]: (1) PPP within the cytoplasm; (2) Folate-mediated one-carbon metabolism in the cytoplasm, mitochondria, and nucleus; (3) NAD kinase in the cytoplasm and mitochondria; (4) Malic enzyme (ME) in the cytoplasm and mitochondria; (5) Nicotinamide nucleotide transhydrogenase (NNT) in the mitochondria; (6) NADP-dependent isocitrate dehydrogenases in the cytoplasm or mitochondria; (7) Glutamine metabolism; (8) Fatty acid oxidation (FAO). The PPP in the cytoplasm is a major contributor to NADPH regulation during disulfidptosis, according to studies [8]. More research is needed to determine the impact of other NADPH homeostasis-regulating pathways on disulfidptosis, this review will primarily focus on the the mechanisms of the PPP.

#### Pentose phosphate pathway (PPP)

Hexokinase converts glucose to glucose-6-phosphate (G6P), which kicks off the PPP process. This pathway is divided into two distinct phases: oxidative and non-oxidative. NADPH is primarily produced during the oxidative phase through three irreversible reactions [9] (Fig. 4):

1. The first key step in the oxidative phase of the Pentose Phosphate Pathway is catalyzed by glucose-6-phosphate dehydrogenase (G6PDH), which oxidizes G6P to 6-phosphogluconolactone while reducing NADP^+^ to NADPH.
2. 6-Phosphogluconolactonase (6PGL) then hydrolyzes 6-phosphogluconolactone, producing 6-phosphogluconate (6PG).
3. 6-Phosphogluconate dehydrogenase (6PGD) catalyzes 6PG’s oxidative decarboxylation, producing ribulose-5-phosphate (Ru5P) and a second molecule of NADPH.

According to research, simulating glucose deprivation is vital for understanding disulfidptosis, with the reduction of NADPH being the most important aspect [8, 10–13]. For example, the GLUT1 inhibitor BAY-876 and the GLUT1/3 inhibitor KL-11,743 cause disulfidptosis in cancer cells that express high levels of slc7a11. They achieve this by inhibiting glucose uptake, which reduces NADPH synthesis through the PPP [8] (Fig. 4). Therefore, the development and exploration of inhibitors that target NADPH-synthesizing enzymes in the PPP is critical. Notable G6PD inhibitors include 6-aminonicotinamide (6-AN), RRx-001, aspirin, and polydatin, among others (Fig. 4). These inhibitors modulate G6PD activity through a variety of mechanisms, affecting NADPH production and contributing to their potential role in cancer therapy [119–123]. However, the hypothesis that these inhibitors induce disulfidptosis in cancer cells by simulating glucose deprivation requires further investigation and validation.

NADPH is crucial not only for cellular energy metabolism, but also for maintaining cellular redox balance. According to studies, elevated NADPH levels produced by the PPP in tumor cells facilitate in their adaptation to oxidative stress. This adaptation allows cancer cells to survive in environments containing high levels of ROS [124]. Consequently, a better understanding of the PPP and its inhibitors has two advantages: it elucidates disulfidptosis mechanisms and opens the door to new therapeutic approaches for cancer and other diseases linked to dysregulated energy metabolism.

### RAC1-WRC pathway

The RAC1-WRC pathway is heavily involved in disulfidptosis, likely functioning at the end of this cell death mechanism [13]. This pathway is activated by the stress of cellular disulphide metabolism. However, its in vivo assembly, the precise mechanism of disulfidptosis regulation, and its potential as a targeted therapeutic approach in specific diseases necessitate further investigation [8, 10–13]. NCK-associated protein 1 (NCKAP1) is an essential component of the WAVE Regulatory Complex (WRC) [125]. Overexpression of RAC1 activates NCKAP1, which leads to the assembly of the WRC with other subunits including WAVE-2, CYFIP1, Abi2, and HSPC300. This complex regulates actin polymerization and lamellipodia formation [126–128]. The RAC1-WRC pathway may contribute to disulfidptosis by activating Arp2/3-mediated lamellipodia formation, which facilitates the formation of disulfide bonds between F-actin filaments. In contrast,, the deficiency of specific WRC subunits has been shown to inhibit disulfidptosis [8, 126]. Although the specific mechanism by which lamellipodia promote disulfide bond formation in disulfidptosis has not been fully elucidated, recent studies have proposed a hypothesis that the Rac-WRC pathway, triggered within lamellipodia, might provide a critical target for the formation of disulfide bonds between actin cytoskeletal proteins through the branched actin network [8]. This finding offers a new perspective on understanding the dynamic regulation of the cytoskeleton and its interaction with protein structural stability, which is significant for in-depth studies of cell signaling and structure-function relationships. Consequently, the regulatory functions of RAC1 and its associated proteins, including NCKAP1, WAVE-2, CYFIP1, Abi2, and HSPC300, may have a significant impact on disulfidptosis.

The direct connection between lamellipodia and disulfide bond formation may not be significant, as they involve different aspects of cellular behavior and molecular stability, respectively. However, there is an indirect connection and mutual influence between them, particularly in the following two aspects. Extracellular matrix (ECM) proteins, such as fibronectin, contain disulfide bonds that are crucial for their structure and function. Lamellipodia interact with these ECM proteins via receptors such as integrins, promoting cell migration and indirectly being influenced by the role of disulfide bonds in maintaining ECM protein structure [129–131]. Within the cell, specific protein-protein interactions are essential for actin polymerization and the dynamic changes of lamellipodia, and these interactions may be influenced by the secondary and tertiary structures of proteins, the stability of which often depends on disulfide bonds [132–134]. Therefore, although lamellipodia and disulfide bond formation may seem independent at a biological level, they are interconnected at multiple levels through a complex network that affects cellular behavior and molecular functional stability, reflecting the interdependence of cellular holistic biological functions and interactions.

#### RAC1

Ras-related C3 botulinum toxin substrate 1 (Rac1), a Rho family small GTPase, acts as an intracellular transducer, orchestrating multiple signaling pathways. These pathways include the control of cytoskeletal structure, transcription, and cell proliferation [135, 136]. Rac1 is localized at the leading edge of migrating cells, and microtubule growth can activate Rac1, promoting the formation of lamellipodia [136]. Rac1 is intricately linked to the formation of the actin cytoskeleton, particularly in the development of lamellipodia, which is thought to be essential for cell motility [137]. Recent research in cells with high SLC7A11 expression has confirmed that the constitutively active Rac1 (Rac1 Q61L mutant) promotes lamellipodia formation and induces disulfidptosis, which is not observed in cells with NCKAP1 knock-out [8]. The interaction of Rac1 with two distinct affinity binding sites (sites A and D) on the WRC may enhance WRC activation, catalyzing actin polymerization through the action of the Arp2/3 complex [138, 139]. Arf1, another WRC activator, works together with Rac1 to stimulate WRC activation. However, the specific mechanism underlying Arf1’s action requires further investigation [140]. Additionally, RAC1 participates in endocytic activities, specifically inducing actin-rich membrane protrusions during macropinocytosis in dendritic cells [141]. A defined region of Rac1 (residues 124–135) is involved in inducing superoxide production in quiescent fibroblasts. This activity serves as a mediator in mitotic signal transduction [142]. Consequently, Rac1 plays an important role in cytoskeletal regulation and signal transduction processes associated with disulfidptosis, particularly in the activation of the WRC being a critical aspect. However, the complexities of the interaction between RAC1 and WRC, as well as their specific targets in the context of disulfidptosis, remain areas ripe for further research.

#### NCKAP1

NCKAP1 has been found in sporadic Alzheimer’s Disease cases and is widely expressed in various cell types in the developing cerebral cortex [143–145]. NCKAP1 is intimately involved in cellular processes such as proliferation and cancer metastasis. It orchestrates motility and adhesion mechanisms at cellular junctions by interacting with OL-protocadherin (OL-pc), which then recruits the NCKAP1-WAVE1 complex to intercellular contact sites [146]. NCKAP1 has been shown to inhibite cancer cell proliferation by arresting the cell cycle at the G2/M phase via the RB1/P53 pathway [147]. Furthermore, NCKAP1 interacts with Heat Shock Protein 90 (HSP90), which promotes cancer cell invasion and metastasis [148]. MiR-214 regulates NCKAP1 in vascular smooth muscle cells, where it is involved in cell proliferation, migration, and neointimal hyperplasia [127]. NCKAP1 interacts with Rac1 and other WRC components, such as WAVE-2, CYFIP1, Abi2, and HSPC300. This interaction is critical in regulating WAVE activity, which is required for Arp2/3-mediated actin polymerization, membrane edge protrusion, and lamellipodia extension. These processes are crucial in the case of disulfidptosis [149, 150]. NCKAP1 can inhibit tumor cell growth via multiple pathways, including ribosomal signaling, oxidative phosphorylation, TGF-β, EMT-related, and RB1/P53 signaling pathways [147, 151]. The potential role of NCKAP1 in promoting disulfidptosis in tumor cells requires further investigation.

#### WAVE-2

WAVE-2, a member of the Wiskott-Aldrich Syndrome Protein (WASP) family that also includes WASP, NWASP (Neural WASP), WAVE-1, and WAVE-3, is primarily responsible for actin cytoskeleton reorganization. Its plays an important role in processes like synaptic remodeling, neurite extension, cell migration, and endocytosis [152]. WAVE-2 is found at the leading edge of lamellipodia, and the amino acid residues 54 to 83 in the WAVE2 SHD are crucial for its specific localization at filopodial tips [153]. WAVE proteins are distinguished by several conserved domains, including the N-terminal WAVE Homology Domain (WHD), the Serine-Histidine Domain (SHD), a proline-rich domain, and the C-terminal verprolin/cofilin/acidic (VCA) domain [154, 155]. Significantly, the VCA region of WAVE proteins interacts with two different protein types to start actin polymerization. The CA domain interacts with the Arp2/3 complex, while the V domain binds to actin monomers (G-actin). This dual binding allows actin monomers be more closely associated with the Arp2/3 complex, thereby promoting efficient actin nucleation [156]. Actin polymerization may play an important role in the formation of disulfide bonds in actin and disulfidptosis.

According to research, the recruitment of WAVE proteins to the membrane occurs through two primary mechanisms: initially, GTP-bound Rac directly interacts with the WAVE complex component Sra1. The proline-rich domain of mammalian WAVE-2 then associates with the SH3 domain of the membrane-bound protein IRSp53, which, in turn, binds to activated Rac [156]. The interaction of activated Rac with Sra1 activates the WRC, potentially leading to a conformational change in the VCA region. This change causes the VCA region to be released from the WRC, which facilitates the activation of the Arp2/3 complex [157]. Moreover, WAVE2 is influenced by several phosphorylation events. For example, c-Abl kinase activates WAVE2 via tyrosine phosphorylation, which promotes actin remodeling in cells. This modulation affects membrane ruffling and cell spreading, with Abi-1 acting as an important link between c-Abl and WAVE2 [158].

The role of WAVE-2 in actin cytoskeletal reorganization is critical for the maintenance of normal cellular functions [152]. WAVE-2 uses its multiple domains to orchestrate the activation of the Arp2/3 complex, which has a significant impact on actin polymerization and the dynamic cytoskeleton reorganization. These processes are vital for cellular physiological activities and may be associated with cell death mechanisms, including disulfidptosis. Notably, the VCA domain of WAVE-2 may regulate disulfidptosis [156].

#### CYFIP1

Cytoplasmic fragile-X mental retardation protein (FMRP)-interacting protein 1 (CYFIP1), also known as Sra1, belongs to the human CYFIP protein family [143]. CYFIP1 has direct interactions with activated Rac1 and F-actin, indicating that it is a Rac1 signaling target [159]. The WRC’s regulatory control relies heavily on CYFIP1. Neurons and lymphoblast-like cells in mice lacking CYFIP1 showed lower mRNA expression levels of WRC components. This finding suggests that CYFIP1’s regulatory influence is primarily at the transcriptional level [160]. CYFIP1 interacts with the FMRP (encoded by the FMR1 gene), but not with the related proteins FXR1P and FXR2P. Notably, the absence of FMRP is closely linked to the pathogenesis of Fragile X Syndrome [143, 161]. Furthermore, the Notch1 signaling pathway regulates CYFIP1 expression. In vitro studies of squamous cell carcinoma (SCC) show that activated Notch1 binds to the CSL sequence in the CYFIP1 gene promoter, upregulating CYFIP1 at both the mRNA and protein levels. This increase could reduce the invasiveness of SCC [162]. CYFIP1 is also involved in axonal growth and formation, transporting the Sra-1/WAVE1 complex to axons via CRMP-2, which is dependent on kinesin-1 [163]. YFIP1 may also modulate cellular responses to both external and internal stressors through its involvement in signal transduction. These stress responses typically engage multiple signaling pathways, including the MAPK and PI3K/Akt pathways, all of which can influence the cellular redox state and the fate of the cell [164–166]. Overall, YFIP1, through its multifaceted roles in cellular signaling, could indirectly affect the redox status of the cell and pathways related to disulfide bond-dependent cell death. The potential impact of CYFIP1 expression, which is regulated by the Notch1 signaling pathway, on the WRC-mediated F-actin regulation and its subsequent influence on disulfidptosis is a compelling area for research [159–162].

#### Abi2

The Abi protein family, which includes Abi1, Abi2, and Abi3, is significantly involved in cytoskeletal reorganization. They are particularly important in processes such as membrane ruffling, cell migration, and lamellipodia formation [167–171]. Abi-2 contains an SH3 domain and a proline-rich sequence, that is required for its interaction with c-Abl. It also serves as a substrate for the c-Abl tyrosine kinase [168]. Abi can directly interact with the WHD of WAVE2, which coordinates the WAVE complex and increases WAVE2’s actin polymerization activity [169]. PIM1 kinase directly phosphorylates ABI2, which regulates its activity. This phosphorylation increases WRC activity, which enhances actin dynamics and as a result, facilitates prostate cancer invasion [170]. Furthermore, ABI2 is involved in other biological pathways. The ABI2-mediated transcription axis MEOX2/KLF4-NANOG promotes tumor growth, metastasis, and sorafenib resistance. This mechanism is essential for maintaining the cancer stem cell (CSC) population, which explains its significance in cancer progression [171]. The WHD of WAVE2 may be involved in the regulation of disulfidptosis. However, the precise domain of interaction between Abi2 and WAVE2 requires further investigation.

#### HSPC300

HSPC300 (Hematopoietic Stem/Progenitor Cell Protein 300) is the WRC’s most conserved subunit, a small 8 kDa protein known for its important role in plant cytoskeletal remodeling [172]^,^ [173]. Nudel, a protein involved in lamellipodia formation, physically interacts with HSPC300, protecting it from proteasome-mediated degradation. Nudel also helps to reduce the instability of certain subunits and subcomplexes within the WRC, which is important during the early stages of WRC assembly [174]. Notably, in vitro, HSPC300 has been shown to directly bind to WAVE2, interact with the N-terminal SHD of Scar/WAVE 1, 2, and 3, and colocalize with all three WAVE proteins in vivo [175, 176]. According to current research, HSPC300, as a vital component of the WRC, is involved in WRC assembly and cytoskeletal remodeling [172–174]. However, the precise role of HSPC300 in the assembly of the WRC and its mechanism in disulfidptosis require further investigation.

Overall, proteins such as RAC1, NCKAP1, WAVE-2, CYFIP1, Abi2, and HSPC300 play important roles in regulating the WRC. They play critical roles in cytoskeletal reorganization, cell migration, and the disulfidptosis mechanism. Current research provides valuable insights into their complex interactions and biological functions. However, much remains to be learned about their specific mechanisms in WRC assembly and disulfidptosis. Future research should concentrate on a thorough examination of these protein interactions and their potential therapeutic implications in disease management. Such research will not only enhance our understanding of fundamental cellular biology but may also reveal novel therapeutic targets for diseases.

## Disulfidptosis and disease

Since the discovery of disulfidptosis, a growing body of research revealed the link between disulfidptosis-related genes and disease prognosis, confirming the importance of novel disulfidptosis signaling pathways in various diseases. This section aims to provide a comprehensive overview of the role of disulfidptosis in cancer, neurodegenerative diseases, and metabolic disorders (Table 1).

**Table 1:** Disulfidptosis-related genes in disease

Disease		Disulfidptosis-related genes
Cancer	Digestive System	1.liver cancer: SPP1, MYBL2 [18]; SLC7A11 [19, 20]; LRPPRC [20]; TNFRSF14, TNFRSF4, TNFSF4 [21], BTN2A1, BTN2A2 [21]; SLCO1B1 [22] 2.CRC: MXRA8, IGFBP5, MRC2, HTRA1, TNFAIP6, SLC3A7, GRP, APOD [23]; TRIP6, OXSM, MYH3, MYH4 [24] 3.ESCC: CD96, SOX17 [25]
	Urinary System	1.BCa: SLC7A11, SLC3A2, RPN1, NCKAP1 [26]; POU5F1, CTSE [27] 2.UC: GYS1, LRPPRC, NDUFA11, OXSM, RPN1, SLC3A2, SLC7A1, TM4SF1 [28] 3.RCC: AJAP1 [29]; ISG20 [30]; MSH3 [31]
	Respiratory System	Lung Cancer: G6PD [32]; OGFRP1 [33]
	Reproductive System	Breast Cancer: CD80, CD276 [34]; DNUFS1, LRPPRC, SLC7A11 [35]
Neurodeenerative diseases		MYH9, IQGAP1, ACTN4, DSTN, ACTB, MYL6, GYS1 [45]
Metabolic diseases		TRXR1, NFATC1 [46]

### Cancer

In the field of disulfidptosis cancer research, current efforts are primarily focused on bioinformatics exploration, which utilizes existing datasets to analyze and predict potential therapeutic targets. This data-driven approach provides important directional guidance for identifying potential targets. However, to validate these predictive outcomes and to gain a deeper understanding of the underlying molecular mechanisms, the next phase of research will require more experimental work. These experiments will help to elucidate the specific connections between disulfidptosis and cancer progression, thereby laying a solid foundation for the development of new therapeutic strategies.

#### Digestive system

SPP1 is an acidic glycoprotein found in many different cell types that plays an important role in tumor cell proliferation, metastasis, invasion, and migration [177]. MYBL2 is a transcription factor that regulates cell proliferation and differentiation, actively participating in the complex processes of the cell cycle [178]. SPP1 and MYBL2 emerge as key genes in disulfidptosis characteristics, suggesting that they may have implications for predicting the survival outcomes of liver cancer patients [18]. The disulfidptosis-related gene SLC7A11 is proposed to potentially emerge as a new prognostic biomarker for hepatocellular carcinoma (HCC), presenting both opportunities and challenges for the development of personalized cancer immunotherapy strategies [19]. Furthermore, studies indicate that disulfidptosis-related genes, specifically SLC7A11 and LRPPRC, could potentially serve as independent prognostic factors for HCC [20]. Immune checkpoint genes, such as TNFRSF14, TNFRSF4, TNFSF4, BTN2A1, and BTN2A2,are suggested to have a strong correlation with disulfidptosis and may play an important role in enhancing tumor immunity, potentially making them useful indicators for predicting the prognosis of HCC [21]. In vitro experiments have shown that the overexpression of the disulfidptosis-related glycolysis gene SLCO1B1 effectively inhibits the proliferation, migration, and invasion of HCC cells. These findings provide valuable reference for developing targeted therapeutic strategies [22]. Li et al. have identified eight characteristic genes associated with disulfidptosis, including MXRA8, IGFBP5, MRC2, HTRA1, TNFAIP6, SLC3A7, GRP, and APOD. These genes are proposed to hold potential as novel biomarkers for colorectal cancer (CRC) [23]. Hu and colleagues have identified four disulfidptosis-related genes, TRIP6, OXSM, MYH3, and MYH4, which are speculated to not only offer potential insights into the survival rates of colorectal adenocarcinoma but might also play a role in influencing the tumor microenvironment, drug sensitivity, and immune landscape [24]. Research indicates that the disulfidptosis-related genes CD96 and SOX17 could potentially act as independent prognostic factors in patients with esophageal squamous cell carcinoma (ESCC) [25]. . In vitro experiments have conclusively demonstrated that downregulating CD96 significantly reduces ESCC cell proliferation. These findings suggest CD96, a disulfidptosis-related gene, as a possible prognostic and therapeutic target for ESCC [25].

#### Urinary system

Bladder cancer is one of the most common cancers of the urinary tract. Chen et al. identified POU5F1 and CTSE as key components in the disulfidptosis-related prognostic model. In vitro studies revealed that POU5F1 transactivates CTSE, promoting the proliferation and metastasis of bladder cancer (BCa) cells. According to these findings, POU5F1 and CTSE could be potential targets for clinical treatment of BCa [27]. Furthermore, a group of disulfidptosis-related genes (GYS1, LRPPRC, NDUFA11, OXSM, RPN1, SLC3A2, and SLC7A1) are thought to influence the prognosis of bladder urothelial carcinoma (UC) via immune cell infiltration [28]. Significantly, knocking down TM4SF1 reduces bladder urothelial carcinoma proliferation, revealing the role of disulfidptosis in bladder urothelial carcinoma and sheds light on its potential regulatory mechanisms [28]. The AJAP1 gene encodes a protein that plays an important role in facilitating cell adhesion and migration [179]. Recent research has revealed a decrease in the expression of the disulfidptosis-related differential gene AJAP1 in clear cell renal cell carcinoma (ccRCC) tissues. This reduction establishes AJAP1 as a novel biomarker for ccRCC and suggests that it may be useful as a therapeutic target for ccRCC patients [29]. Peng and colleagues identified a link between ISG20 and disulfidptosis, as well as tumor immunity, indicating its potential as a therapeutic target for ccRCC [30]. . MSH3 emerges as a essential gene in the cellular process of disulfidptosis, as demonstrated by in vitro experiments. Overexpression of MSH3, particularly under conditions of glucose starvation and concurrent SLC7A11 overexpression, has been demonstrated to promote disulfidptosis in renal cell carcinoma (RCC) cells [31]. Interestingly, we found that Zhao and colleagues have identified four disulfidptosis-inhibiting genes (SLC7A11, SLC3A2, RPN1, and NCKAP1). These genes are implicated in playing pivotal roles in the development and immune infiltration processes of bladder cancer [26]. However, previous studies have demonstrated that SLC7A11 and NCKAP1 play a significant role in inducing disulfidptosis. The contradictions in these findings may be attributed to the specificity of tumor cells, the uniqueness of different databases, and the variations in bioinformatics analysis algorithms.

#### Respiratory system

The significance of G6PD in lung adenocarcinoma has been highlighted due to its association with poor prognosis and pro-carcinogenic effects. Treatment with G6PD inhibitors has been shown to effectively reduce G6PD expression in lung cancer cells, resulting in a significant inhibition of their proliferative capacity. This inhibitory effect is thought to be achieved by reducing NADPH production, which induces disulfidptosis [32]. Yang et al. discovered a strong link between the long non-coding RNA (lncRNA) OGFRP1 and disulfidptosis-related genes. Inhibiting OGFRP1 through experimental interventions significantly reduces lung cancer cells’ invasion and migration capabilities. These findings suggest a potential regulatory role of disulfidptosis in lung cancer [33].

#### Reproductive system

Wang and colleagues discovered a link between disulfidptosis-related immune checkpoint hub genes (CD80 and CD276) and the onset, progression, and mortality of breast cancer. These findings suggest new possibilities for studying immunotherapy in the context of breast cancer^30^. Wang and colleagues also discovered three disulfidptosis-related genes, DNUFS1, LRPPRC, and SLC7A11, with differential expression and prognostic values. These findings offer important insights into predicting the prognosis of breast cancer patients [35].

### Neurodegenerative diseases

Alzheimer’s disease(AD) is a common neurodegenerative disorder characterized by a progressive decline in cognitive function, decreased daily living abilities, and the emergence of psychiatric symptoms [180]. A study identified seven genes associated with disulfidptosis (MYH9, IQGAP1, ACTN4, DSTN, ACTB, MYL6, and GYS1). These genes can not only accurately assess AD subtypes, but also diagnose AD patients, providing new insights into the disease’s potential pathogenesis and treatment strategies [45].

### Metabolic diseases

Overactivation of osteoclasts is closely linked to diseases, including rheumatoid arthritis, osteoporosis, and bone metastases [181–183]. Mature osteoclasts in these diseases help to destroy bone through resorption. As a result, inhibiting osteoclast differentiation has emerged as a successful treatment strategy, as evidenced by basic research and clinical trials. A recent study proposed that NFATC1-mediated expression of SLC7A11 sensitizes osteoclast precursors to TRXR1 inhibitors, resulting in intracellular cystine accumulation and disulfidptosis. This novel approach selectively targets osteoclast precursors, which inhibits bone resorption [46].

## Conclusion

Disulfidptosis, a newly defined type of cell death, has distinct characteristics [8, 10–13]: (1) An abnormal intracellular accumulation of disulfides, including cysteine; (2) Depletion of NADPH, which can be caused by glucose scarcity and H_2_O_2_ exposure; (3) Atypical disulfide bonds are formed within actin cytoskeletal proteins; (4) Disulfide reductants or agents that regenerate free thiols via disulfide exchange can prevent cell death; (5) Resistance to rescue by ROS scavengers (Trolox) or inhibitors of alternative cell death pathways (apoptosis and ferroptosis).However, the exact mechanisms of disulfidptosis still remain to be further elucidated. The involvement of actin cytoskeletal proteins has been determined based on changes in the disulfide bond proteome [8]. The reasons for the selective vulnerability of actin cytoskeletal proteins to disulfide stress remain elusive, as does the potential involvement of additional cytoskeletal proteins, including those associated with microfilaments, microtubules, and intermediate filaments, in the initiation of disulfidptosis.

SLC7A11 primarily regulates the transport of extracellular cystine into cells. Its increased expression gives cancer cells an advantage by increasing GSH synthesis and inhibiting ferroptosis caused by oxidative stress [47–49]. Conversely, during glucose deprivation, cancer cells with high SLC7A11 expression undergo cystine accumulation, resulting in disulfide stress. This stress promotes the formation of disulfide bonds in actin, which results in disulfidptosis [8, 47]. Thus, disulfidptosis and ferroptosis may be inextricably linked via SLC7A11 expression: high SLC7A11 levels inhibit ferroptosis but induce disulfidptosis under glucose deprivation, whereas low SLC7A11 expression promotes ferroptosis but does not induce disulfidptosis. The precise mechanisms underpinning these processes, however, still require further investigation.

In addition to xCT, cystinosin has been identified as another cystine transporter capable of facilitating cystine translocation from the lysosome to the cytoplasm [66]. Transporters such as ASCT1/2 and EAAT3 play an important role in cellular physiology by directly mediating cysteine uptake from the extracellular milieu into the intracellular environment [60, 79]. Contemporary focuses primarily on cells that express high levels of SLC7A11. However, it is critical to investigate whether cystine accumulations, facilitated by alternative pathways, could similarly cause disulfidptosis. Investigating the potential role of other cystine transporters, such as cystinosin, and cysteine transporters, in cystine accumulation-induced disulfidptosis is an important area of research.

Disulfidptosis is thought to require glucose deprivation [8, 10–13]. Recent studies, however, suggests that the cell death observed in SLC7A11-overexpressing cells, which is caused by NADPH depletion in response to H_2_O_2_ exposure, may be a variant of disulfidptosis [117]. Consequently, it remains unclear whether alternative routes of NADPH depletion, particularly through the inhibition of enzymes such as G6PDH, ME, IDH1, IDH2, and those involved in folate metabolism, facilitate disulfidptosis in cells with increased SLC7A11 expression. This area requires further investigation to elucidate these potential connections.

We hypothesize that the cystine accumulation and subsequent disulfide stress will activate the RAC1-WRC signaling pathway, which is regarded as a essential mechanism in disulfidptosis [13]. Overexpression of RAC1 activates NCKAP1, which then orchestrates the assembly of WAVE-2, CYFIP1, Abi2, and HSPC300, resulting in the formation of the WRC. This complex, in turn, activates the Arp2/3 complex, which promotes lamellipodia formation. This process is necessary for the formation of disulfide bonds within F-actin, which contributes to the progression of disulfidptosis [8]. However, our understanding of the WRC’s assembly mechanisms in vivo, the regulatory roles of its individual components in disulfidptosis, and the specific mechanisms by which cystine accumulation facilitates disulfide bond formation within F-actin remain elusive. These aspects represent critical knowledge gaps that require extensive further investigation.

Despite significant achievements in this field in recent years, a range of unresolved questions and challenges remain. In this section, we will systematically outline these challenges, opening up potential future research directions:1. Investigate whether abnormal cystinosin activation or cysteamine application influences the cytoplasmic accumulation of cystine, potentially triggering disulfidptosis;2. Examine whether the overactivation of ASCT1/2 and EAAT3 under the influence of thiol oxidants can mimic the accumulation of cystine observed with high expression of SLC7A11, thereby promoting the occurrence of disulfidptosis; 3. Explore whether other pathways of NADPH depletion, such as inhibition of G6PDH, ME, IDH1, IDH2, and enzymes related to folate metabolism, also promote disulfidptosis in SLC7A11-high cells; 4. Investigate whether SLC7A11-high cells, which exhibit resistance to ferroptosis, become susceptible to disulfidptosis with the depletion of NADPH; 5. Elucidate the in vivo assembly mechanism of the RAC1-WRC pathway and how its various components interact to influence the process of disulfidptosis.

This article provides a comprehensive review of current advances in disulfidptosis research, including its historical discovery, molecular complexities, regulatory mechanisms, protein pathways, and disease implications. Additionally, we propose potential signaling pathways that could influence disulfidptosis. These findings not only contribute new insights for future research into disulfidptosis but also present novel disease treatment strategies. Future research should focus on the specific functions and mechanisms of disulfidptosis-associated genes, providing comprehensive insights for the prevention, diagnosis, and treatment of diseases.

## Data Availability

N/A.

## Abbreviations

SLC7A11: Solute carrier family 7 member 11
PPP: Pentose phosphate pathway
PCD: Programmed cell death
ACD: Accidental cell death
NADPH: Nicotinamide adenine dinucleotide phosphate
WRC: WAVE regulatory complex
SLC: Solute carrier
GSH: Glutathione
GPX4: GSH-dependent glutathione peroxidase 4
ROS: Reactive oxygen species
ASCT1/2: Alanine-Serine-Cysteine transporters 1 and 2
EAAT: Excitatory amino acid transporter
Trx: Thioredoxin
TrxR: Thioredoxin reductase
AF: Auranofin
TRi-1: TRXR1 inhibitor 1
2DG: 2-deoxyglucose
DTT: Dithiothreitol
2ME: β-mercaptoethanol
TCEP: tris(2-carboxyethyl)phosphine
NAC: N-acetylcysteine
L-Pen: L-penicillamine
γ-GCS: γ-glutamylcysteine synthetase
GS: Glutathione synthetase
LOOH: Lipid hydroperoxides
LOH: Lipid alcohols
GSSG: Oxidized glutathione
GR: Glutathione reductase
H_2_O_2_: Hydrogen peroxide
ME: Malic enzyme
NNT: Nicotinamide nucleotide transhydrogenase
FAO: Fatty acid oxidation
G6P: Glucose-6-phosphate
G6PDH: Glucose-6-phosphate dehydrogenase
6PGL: 6-Phosphogluconolactonase
6PG: 6-phosphogluconate
6PGD: 6-Phosphogluconate dehydrogenase
Ru5P: Ribulose-5-phosphate
6-AN: 6-aminonicotinamide
GLUT1/3: Glucose transporter 1/3
NCKAP1: NCK-associated protein 1
Rac1: Ras-related C3 botulinum toxin substrate 1
WASP: Wiskott-Aldrich syndrome protein
WHD: WAVE homology domain
SHD: Serine-Histidine domain
VCA: Verprolin/cofilin/acidic domain
CYFIP1: Cytoplasmic fragile-X mental retardation protein (FMRP)-interacting protein 1
SCC: Squamous cell carcinoma
CSC: Cancer stem cell
HCC: Hepatocellular carcinoma
CRC: Colorectal cancer
ESCC: Esophageal squamous cell carcinoma
BCa: Bladder cancer
ccRCC: Clear cell renal cell carcinoma
RCC: Renal cell carcinoma
AD: Alzheimer’s disease
