# Correction: Disulfidptosis decoded: a journey through cell death mysteries, regulatory networks, disease paradigms and future directions

**DOI:** 10.1186/s40364-025-00761-7

**Published:** 2025-03-11

**Authors:** Jinyu Chen, Boyuan Ma, Yubiao Yang, Bitao Wang, Jian Hao, Xianhu Zhou

**Affiliations:** The Second Affiliated Hospital, Guangzhou Medical University, Guangzhou, 510260 China

**Biomarker Research (2024) 12:45**

10.1186/s40364-024-00593-x

The authors note that in the Funding section of the original manuscript is erroneously listed the grant number No. 81371957 which instead corresponds to a project that concluded in 2017.

The authors wish to clarify that the correct grant details are as follows:

National Natural Science Foundation of China. Number: 82172409.

